# Building dementia knowledge globally through the Understanding Dementia Massive Open Online Course (MOOC)

**DOI:** 10.1038/s41539-019-0042-4

**Published:** 2019-04-10

**Authors:** Claire Eccleston, Kathleen Doherty, Aidan Bindoff, Andrew Robinson, James Vickers, Fran McInerney

**Affiliations:** Wicking Dementia Research and Education Centre, University of Tasmania, Hobart, Australia

## Abstract

The prevalence of dementia is escalating world-wide and knowledge deficits remain a barrier to community inclusiveness and quality care. The need for quality, comprehensive education has been identified as a key priority for global action plans on dementia. The Understanding Dementia Massive Open Online Course (UDMOOC) offers the potential to improve dementia knowledge globally. Completion rates for the UDMOOC (2016–2017) were on average 42% of enrolments, and 69% of participants care or have cared for people with dementia. The current study shows baseline dementia knowledge was positively related to previous learning about dementia from various types of exposure to the condition including having family members and/or working with people with the condition, and having undertaken dementia education. However, knowledge of all participant groups showed substantial improvements after completion of the UDMOOC. This was shown regardless of educational background and previous experience of dementia, and group differences after completing the UDMOOC were minimised. The UDMOOC is therefore an effective knowledge translation strategy to improve dementia knowledge for a diverse, international learner group.

## Introduction

The number of people living with dementia globally is now approaching 50 million, with a projected tripling of that number predicted by 2050. This is largely attributable to anticipated increases in lower and middle income countries as their populations age.^1^ While both the death rate and disease burden from dementia are increasing, rates of diagnosis remain low^2^ and stigma and perceived futility of diagnosis continue to impact on health seeking behaviour.^3^ The aged care workforce is inadequately resourced and prepared to meet the growing need for dementia care,^4^ and dependency on informal community care is increasing,^5^ with a predominance among both informal and paid carers for people with dementia of women possessing lower levels of education.^1^ Levels of knowledge about dementia are inadequate in informal carers^6^ and health care professionals,^7–9^ and are poor to moderate in the general population, particularly those from low-socioeconomic groups.^10,11^ Raising dementia awareness and understanding is a core component of most national dementia policies, with the aim of addressing the persistent misconceptions and associated stigma that remain about dementia.

Knowledge is one dimension of health literacy which underpins an individual’s capacity to interact productively with health care systems and make critical decisions about their health and the health of those for whom they care.^12^ Navigating these complex systems provides a basis on which health literacy can be built,^13^ with knowledge evolving as care needs develop over the trajectory of dementia, increasing demands on the carer and requiring different services. Family carers learn largely by trial and error, active information seeking, application of previous knowledge or skills and being guided by others.^14^ Similarly, unregulated care workers/assistants,^15^ as well as health care students,^16^ gain knowledge through experience, in addition to more formal training opportunities.

Massive open online courses (MOOCs) emerged as a concept in 2008 and developed considerable momentum from 2012. They were purported to bring free, online content, in an accessible format on a wide range of topics, to large communities of learners. While MOOCs have been publicised as a way to provide education to those who would otherwise not have access, they are yet to deliver on their disruptive, emancipatory and democratising potential.^17,18^ The Understanding Dementia MOOC (UDMOOC^19^) aims to improve knowledge of dementia in a broad international community. Over 9 weeks, it focuses on basic neurobiology, dementia pathophysiology, medical management and person-centred care. The UDMOOC was designed to maximise accessibility for non-traditional adult learners who may have low levels of previous education. To that aim, its design includes a conversational framework,^20^ video discussions, summaries and transcripts, discussion forums, games and quizzes.^21^ The UDMOOC is designed to support participants’ understanding of the brain pathology associated with dementia and how this can affect the person with the condition. In this way, it helps carers for people with dementia to comprehend and respond to behaviour in the context of underlying pathology. Completion rates of early iterations of the UDMOOC were relatively high (38–39%) regardless of the participant’s entry level of education.^22,23^

We assessed the effectiveness of the UDMOOC in educating people about dementia in a broad international community by assessing knowledge in a pre-post design over 2 years, using a validated scale, the Dementia Knowledge Assessment Scale,^24,25^ which comprises 25 items considered essential aspects of dementia knowledge. We hypothesised that exposure to both the lived experience of and theoretical understandings about dementia via dementia-specific education, having a family member with the condition or caring for people with dementia in the workforce would be associated with higher pre-intervention knowledge. We also hypothesised that all groups would show higher knowledge after the educational intervention.

## Results

### Demographics

A total of 4894 participants of the 2016 and 2017 UDMOOCs completed the Dementia Knowledge Assessment Scale (DKAS 2.0)^24,25^ before and after the course (Table 1); representing 10% of UDMOOC enrollees. 20,061 (117 countries) and 29,039 (132 countries) participants enroled in 2016 and 2017, respectively. Australians represented 66.5% of all enrolments, and the top non-Australian countries by enrolments included the UK (9.8%), New Zealand (7%), Canada (5.9%), Ireland (2.7%), the US (2.6%), the Philippines (0.7%), Singapore (0.7%), India (0.6%), China (0.3%) and Malaysia (0.2%). The completion rate was 42.01% (±0.53%). Participants of both the UDMOOC and DKAS comprised a high proportion of mid-aged females with current or previous experience providing professional or family care to people with dementia, nearly half without a university-level qualification (Table 1). Median age was 39.0 (interquartile range (IQR): 29.4–48.0) for those completing the DKAS, compared with 47.0 (IQR: 34.0–56.0) for all UDMOOC participants.

**Table 1:** Demographic characteristics of DKAS (Dementia Knowledge Assessment Scale) participants (*n* = 4894) and active participants of the UDMOOC (*n* = 27,265)

Sample characteristics	All UDMOOC participants *n*	All UDMOOC participants %	DKAS participants *n*	DKAS participants %
Female	24,392	89.5	4382	89.9
Male	2824	10.4	491	10.1
Resides in Australia	19,564	71.8	3638	74.3
Nurse	5921	21.7	1085	22.2
Care worker	4928	18.0	797	16.3
Other health worker	3922	14.4	763	15.6
Other occupation	5951	21.8	1097	22.4
Did not provide occupation	6543	24.0	1152	23.5
HLE—High school and below	4106	15.1	539	11.0
HLE—Pre-tertiary	8025	29.4	1752	35.8
HLE—Bachelors degree	8450	31.0	1554	31.8
HLE—Postgraduate/Honours	4748	17.4	1049	21.4
Unpaid carer for a person with dementia	718	2.6	137	2.8
Provided professional care for people with dementia	17,655	64.8	3124	63.8
Both unpaid carer and provided professional care	353	1.3	59	1.2
Have never cared for a person with dementia	8539	31.3	1574	32.2
Previous dementia education	5804	21.3	1003	20.5
Family member diagnosed with dementia	8498	31.2	1638	33.5

*HLE* Highest Level of Education

### Baseline scores

The median score at baseline was 34.5 (IQR: 27–41) out of a maximum possible score of 50. There was a broad pattern of increasing baseline scores associated with increasing levels of dementia-related exposure (*F*_(7,4882)_ = 101.5, *p* < 0.001), and general education (*F*_(4,4882)_ = 131.2, *p* *<* 0.001). Participants with family only exposure to dementia obtained a median baseline score of 28 (IQR: 22–35), which was only marginally better than participants with no exposure to dementia (median = 27, IQR: 20–34). In contrast, participants with work exposure or dementia education obtained median baseline scores of 34 (IQR: 26–40) and 35 (IQR: 29–40), respectively. The greatest median baseline scores were obtained by participants who were in all three exposure categories (median = 38, IQR: 32–43). Similar ranges were observed over general education categories, with those in the “high school and below” education category obtaining a median baseline score of 30 (IQR: 24–37) and those in the “honours and postgraduate” category obtaining a median baseline score of 36 (IQR: 28–42).

### Post-UDMOOC scores

**Fig. 1:**
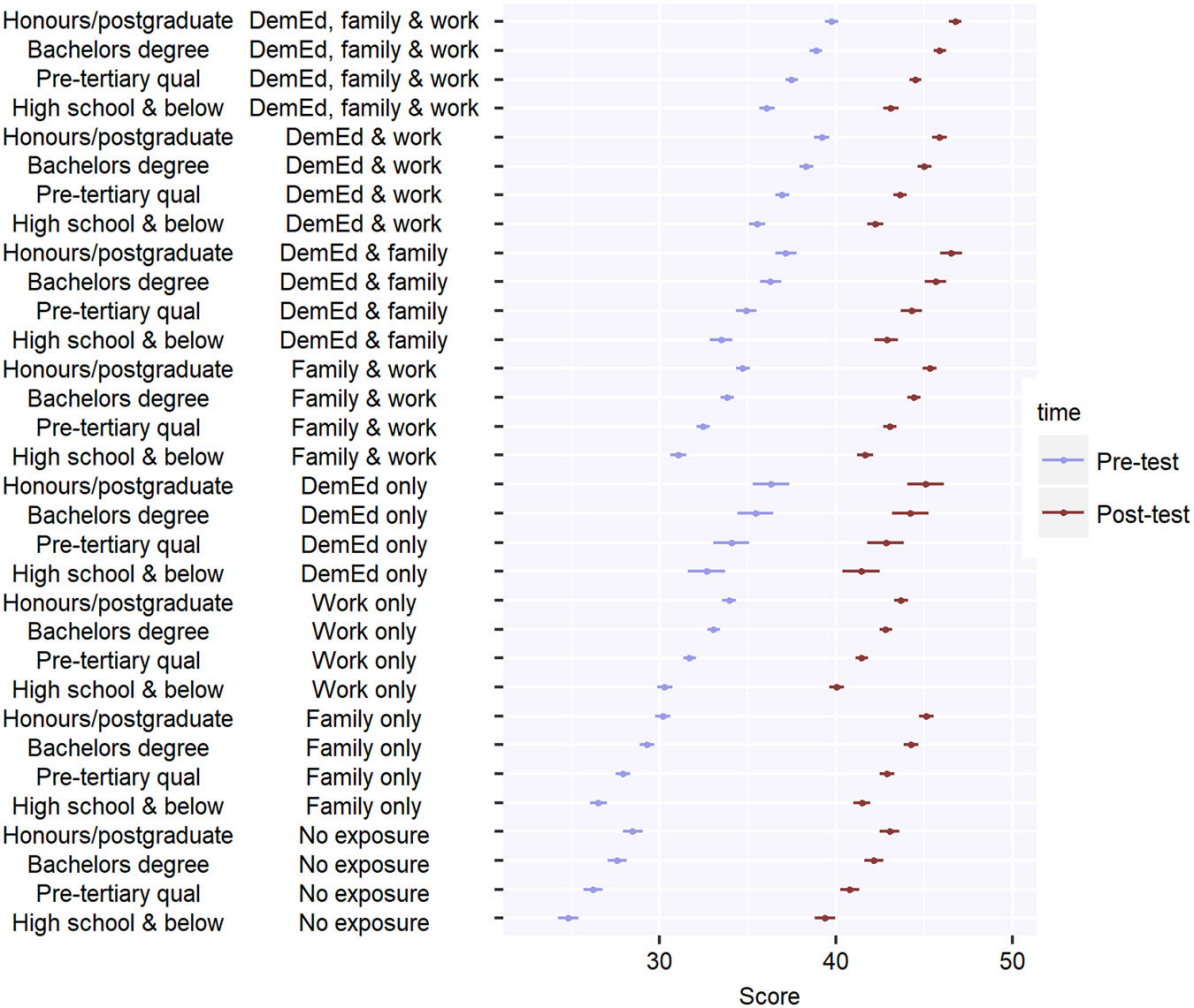
Estimated mean DKAS (Dementia Knowledge Assessment Scale) scores and 95% confidence intervals for 4894 UDMOOC participants with all combinations of educational attainment and dementia-related exposure. Dementia-related exposure is defined by having a family member with dementia (family), having completed dementia-specific education (DemEd), or having worked with people with dementia (work)

The median score post-UDMOOC was 45 (IQR: 41–48), which was a significant increase on baseline scores (*F*_(1,4886)_ = 4245.6, *p* < 0.001 after adjusting for general education and dementia exposure). Participants with the least exposure to dementia obtained the greatest increases in dementia knowledge scores (after adjusting for general education) after taking the UDMOOC. This UDMOOC × exposure interaction was significant (*F*_(7,4886)_ = 87.8, *p* < 0.001), and suggests an equalising capacity for the UDMOOC to increase dementia knowledge across exposure cohorts (see Fig. 1 for further illustration).

**Fig. 2:**
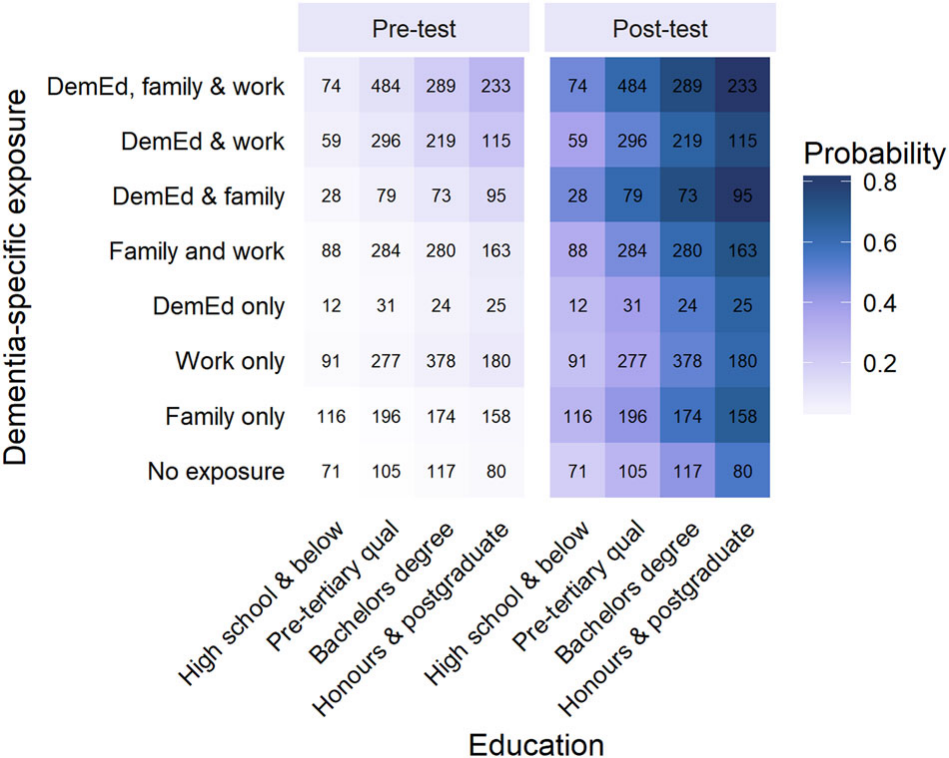
Estimated probabilities of achieving a DKAS (Dementia Knowledge Assessment Scale) score of 45 out of a possible 50 or greater for each combination of dementia-specific exposure and educational attainment before and after undertaking the UDMOOC. Sample size for each combination is shown (total *n* = 4894)

In order to assess the determinants of obtaining a high-DKAS score, a multilevel logistic regression model was fitted which estimated the probability of having a score within a target range (45–50) for education and exposure categories at baseline and post-UDMOOC. Prior to analysis, the 90th percentile of baseline DKAS scores was chosen to represent a very high level of dementia knowledge in the community for people who had not taken the UDMOOC previously. This score was calculated to be 45, 5 points off the maximum achievable score. Similar arbitrary cutoffs have been applied elsewhere to examine the population distribution of scores in educational settings.^26,27^ The probability of achieving this target score was estimated for each exposure and education combination and is presented in Fig. 2.

Both before and after the UDMOOC, higher levels of educational attainment and higher levels of exposure to dementia were associated with higher probability of achieving the target score. Before the UDMOOC, those with no or single types of exposure to dementia had low probabilities of achieving the target score compared with those with two or three types of exposure, particularly those with formal dementia education (Fig. 2), supporting the contention that the type, and number of types, of exposure to dementia are important for dementia knowledge. After completing the UDMOOC, those with no exposure, or family exposure only, had low probabilities of having a target score relative to those with family exposure and dementia education (with and without occupational exposure).

However, the likelihood of obtaining a target score after UDMOOC completion was significantly higher for all groups ($\chi _4^2 = 2223.6$, *p* < 0.001). Examining the two predictor variables together, those with a maximum of high school education and no previous exposure to dementia had the lowest probability of having a target score (Fig. 2), which at pre-test was 0.006 (95% CI = [0.003–0.011]), and at post-test was 0.19 (95% CI = [0.16–0.24]). Highest probability of a target score was for those with honours/postgraduate education and all three identified types of dementia exposure; 0.29 (95% CI: [0.26–0.33]) at pre-test and 0.83 (95% CI: [0.80–0.85]) at post-test. At post-test, 2567 (52%) of participants achieved the target score, compared with 555 (11%) participants at pre-test, clearly demonstrating the potential of this mode of online learning to increase levels of dementia knowledge across broad sections of the community to levels observed in the most knowledgeable segment.

## Discussion

This study examined changes to dementia knowledge associated with completing the Understanding Dementia MOOC, and further, the role of experiential learning and dementia education in the acquisition of this knowledge about dementia. Participants in the UDMOOC ranged from those with limited personal or other experience of dementia, through to domain ‘experts’ possessing considerable personal and occupational experience of dementia and formal dementia education or training. We demonstrated that exposure to the lived experience of dementia through family and work settings, and/or to education about dementia, were associated with higher baseline knowledge of dementia, most markedly in those who had undertaken dementia education but also for those caring for people with dementia in the workforce and to a lesser degree those with a family member with dementia. Experience from multiple different settings was associated with the highest baseline levels suggesting a cumulative effect on knowledge, yet despite this, baseline knowledge was typically not comprehensive, even where all three opportunities for learning were available.

Undertaking the Understanding Dementia MOOC, however, was related to considerably higher knowledge scores and, importantly, reduced the magnitude of knowledge differences associated with different levels of exposure. This demonstrates that the UDMOOC can complement participants’ existing understanding of dementia irrespective of its source, resulting in comprehensive knowledge regardless of experience.

To date there has been limited evidence that MOOCs are an effective knowledge enhancement mechanism. While there has been much emphasis on design and learner attributes, learning outcomes associated with MOOCs are rarely examined (although see^28–30^). Few MOOCs, include objective measures of knowledge acquisition,^30^ instead choosing primary outcome measures such as engagement, retention and completion as evidence of success.^31^ However, not only was the UDMOOC designed to build dementia knowledge in a broad learner cohort,^21^ but validated and robust measures of dementia knowledge were inbuilt to assess whether this occurred.

The significance of demonstrating knowledge improvement across a wide range of participants via a free, widely available online course is particularly evident for dementia. The condition is increasingly prevalent across a globally ageing population, and there is no effective therapeutic intervention currently available.^32^ People with the condition are cared for in the home and workforce by family and paid caregivers, typically women, many of whom may have a limited capacity to effectively participate in adult learning due to low levels of education, low income and the time and energy required for caregiving.^33^ The knowledge of caregivers, health professionals and the general public about dementia is consistently reported as inadequate.^4,7,10,11^ The critical need to address such lack of awareness and understanding about dementia is identified by international public health advocates, including to meet the aims of the Global action plan on dementia.^34,35^ The current data indicate that the UDMOOC is effective in improving awareness and understanding of dementia, across groups including informal carers, care workers, health professionals and the general public.

The important contribution of experiential learning, that is, the construction of knowledge and meaning from real-life experience,^36^ is evident in shaping dementia knowledge. While there will be considerable heterogeneity in the nature and extent of various types of exposure to dementia (education, and family and/or workplace care), each of these contribute to the breadth of understanding about the condition. We suggest that while experiential learning may provide knowledge in some areas, it rarely addresses all of the relevant domains of knowledge, as shown by the low probability of participants with some exposure to dementia achieving the target score at baseline. However, the UDMOOC has demonstrated capacity to increase knowledge across all areas; to bring learners to a more comprehensive level of understanding. Further, despite the evident impact of previous educational experience in shaping people’s knowledge of this health condition, the equalising effect of the UDMOOC is further demonstrated by the large increases in knowledge shown for all educational groups, including that of participants without a tertiary education.

One of the key arguments for MOOCs is their democratising potential.^37,38^ Not only does the probability of having a comprehensive knowledge of dementia greatly increase after completing the UDMOOC, regardless of educational and experiential backgrounds of participants, but the demographics of participants indicate that the UDMOOC is being accessed by the groups that most need it. UDMOOC course participants comprise a high proportion of mid-aged females, around half without a university-level qualification; notably different from the typical educationally advantaged, male participants of MOOCs described by Stich and Reeves.^39^ Most of these care for someone with dementia through their occupation and/or home. While completion rates are insufficient as a measure of educational outcome, it is nonetheless also important to note that these groups are just as likely to complete the UDMOOC^22^ and completion rates for this MOOC since inception have substantially exceeded the typical 5–15% MOOC completion rate.^23^

These figures demonstrate the motivation of the cohort, suggesting the recognition of those most involved in dementia care of the importance of this education and the need for access to evidence based quality education about dementia in order to address the scale of the public health challenge that dementia presents. The global adoption and potential of this education is also evident, with the UDMOOC having been undertaken by participants in more than 180 different countries incorporating individuals of all ages and all levels of previous educational attainment.

In light of difficulties in MOOCs reaching underserved communities,^39^ and little evidence of the substantive educational impact of MOOCs in general^31,40^ and health-related MOOCs in particular,^41^ these data demonstrate the UDMOOC significantly improves dementia knowledge within a short-time frame for a broad range of individuals with a spectrum of exposure to the condition.

This course is an effective mechanism to educate a broad international cohort of informal carers, health care providers and the general public about dementia. This allows a fundamental requirement of the global action plan on dementia to be realised.^35^ In addition, it supports the ability of individuals to more effectively make decisions about their own care needs and those of the people for whom they care, so that as prevalence escalates in the community, people living with dementia will receive better quality care.

## Methods

### Intervention

The Wicking Dementia Research and Education Centre’s Understanding Dementia MOOC was delivered as a 9 week long, free online course in 2016 and again in 2017. The MOOC was advertised in print and online media, through direct mailouts to health and community organisations, social media and through word of mouth. Enrollment was open to anyone. Participants were given the opportunity to complete a pre-course survey before course content was delivered, and to complete the same survey again after completing the final unit of the course.

### Participants

Respondents were participants in either the single offering of the UDMOOC in 2016 or 2017 (the intervention). There were no exclusion criteria. Data were only included in the analysis for those participants who submitted the pre-test survey before accessing any content, for those who completed the survey both at the start and end of the intervention (*n* = 1701 out of 11,706 UDMOOC participants in 2016, and *n* = 3193 out of 15,559 participants in 2017, where UDMOOC participants were defined as those who actively participated in the course), and those who completed the MOOC before post-test. Incomplete cases were excluded from analysis.

Informed consent of the participants was obtained using the Terms and Conditions and Information Sheet, which provided detailed information about the meaning of participation in research and participants were able to select a button to agree to participate or not. They were provided with advice that they could withdraw at any stage and that they were not obligated to complete the survey. The research complied with all relevant ethical regulations and was approved by the Tasmanian Social Science Human Research Ethics Committee (ref: H0013532).

### Measures

DKAS 2.0^24,25^ was used to assess dementia knowledge pre- and post-UDMOOC. This reliable, validated survey comprises 25 items about the characteristics and trajectory of dementia, risk factors and aspects of caring for people with the condition. The items were developed from the results of a Delphi study involving international experts in clinical care and dementia education who identified the essential facts to know about dementia.^42^ Response options, include true, possibly true, false, possibly false and don’t know, which are re-scored to fully correct (2), partly correct (1) or incorrect (0) and summed to create a score from 0 to 50.

Participants also answered a number of demographic questions, including level of completed education, whether they had previously completed any dementia-related education, whether they had a family member diagnosed with dementia, and whether they had previously worked with people with dementia.

### Statistical analysis

Descriptive analyses were conducted, and median DKAS scores are reported as a measure of central tendency due to the non-normal distributions of post-UDMOOC data. Two related inferential analyses were completed, the first examining test scores and the second examining the probability of obtaining a minimum target score defined by the 90th percentile DKAS score at pre-test (45–50 in this cohort). We investigated differences in the distribution of test scores for participants from different dementia-related exposure and formal education categories at pre-test and post-test (before and after UDMOOC exposure). Multilevel models were fitted with random intercepts for each participant to account for the non-independence of observations from pre-test to post-test. A random intercept was also fitted for UDMOOC iteration, in order to account for any variance attributable to year. Modelling the variance attributable to individual participants and UDMOOC iteration enabled the intervention effect (UDMOOC exposure) to be estimated with greater precision (by integrating out variance attributable to random effects), and allowed for the assumption of independence of observations to be relaxed.

The probability of obtaining a desirable score at pre-test and post-test was estimated for each exposure group (adjusting for education) and each education group (adjusting for exposure) using a logistic regression model. Mean scores for the same categories were estimated using a linear regression model. Both models were fitted with the ‘lme4ʼ package in R^43,44^ using restricted maximum likelihood estimation.

Due to violations of distributional assumptions (for the linear model) and inhomogeneous variance (determined using graphical diagnostic methods), 95% confidence intervals were estimated using semi-parametric bootstrapping with 10^4^ iterations. *F* statistics and degrees of freedom were estimated using the conservative Kenward–Roger method of approximation implemented in the ‘pbkrʼ R package.^45^ Chi-squared statistics for the logistic regression model were determined by likelihood ratio test.

Differences between groups were estimated using Tukey contrasts, and two-sided *p* values were adjusted to correct for multiple comparisons using the Tukey method. Estimated differences between education groups were averaged over levels of exposure and vice versa.

### Code availability

All code is available at https://github.com/ABindoff/udmooc_dkas. 10.5281/zenodo.2553707.

### Reporting summary

Further information on experimental design is available in the Nature Research Reporting Summary linked to this article.

## Supplementary information

Reporting Summary

## Data Availability

Data are available from https://github.com/ABindoff/udmooc_dkas.^46^

## Supplementary information

**Supplementary Information** accompanies the paper on the *npj Science of Learning* website (10.1038/s41539-019-0042-4).
